# Supplementary material for: Mutations in CFAP43 and CFAP44 cause male infertility and flagellum defects in Trypanosoma and human
Source: Nat Commun. 2018 Feb 15;9:686. doi: 10.1038/s41467-017-02792-7 (PMC5814398; doi:10.1038/s41467-017-02792-7)
Supplement: Supplementary file 1 — Supplementary Information [file 41467_2017_2792_MOESM1_ESM.pdf]

SUPPLEMENTARY INFORMATION

SUPPLEMENTARY FIGURES

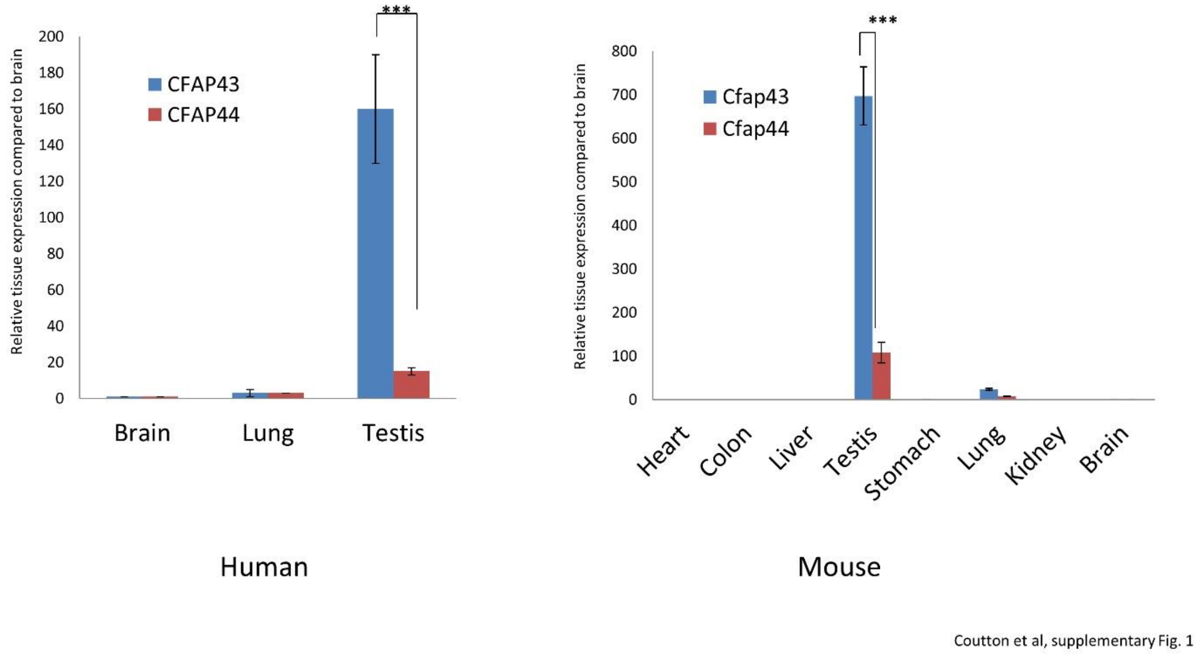

Supplementary Figure 1. Relative mRNA Expression of human and mouse CFAP43 and CFAP44 transcripts. *CFAP43* and *CFAP4* mRNA levels in a panel of human and mouse normal tissues. Results are presented as the mean of triplicates (ratio target gene/ACTB) ± Standard Deviation (SD). RT-qPCR data were normalized using the reference gene ACTB with the -ΔΔCt method. Brain expression is arbitrarily set to 1. In human and mouse, *CFAP43* (blue columns) and *CFAP44* (red columns) have their strongest expression in testis compared to other organs. Unpaired t-test, ***P< 0.001.

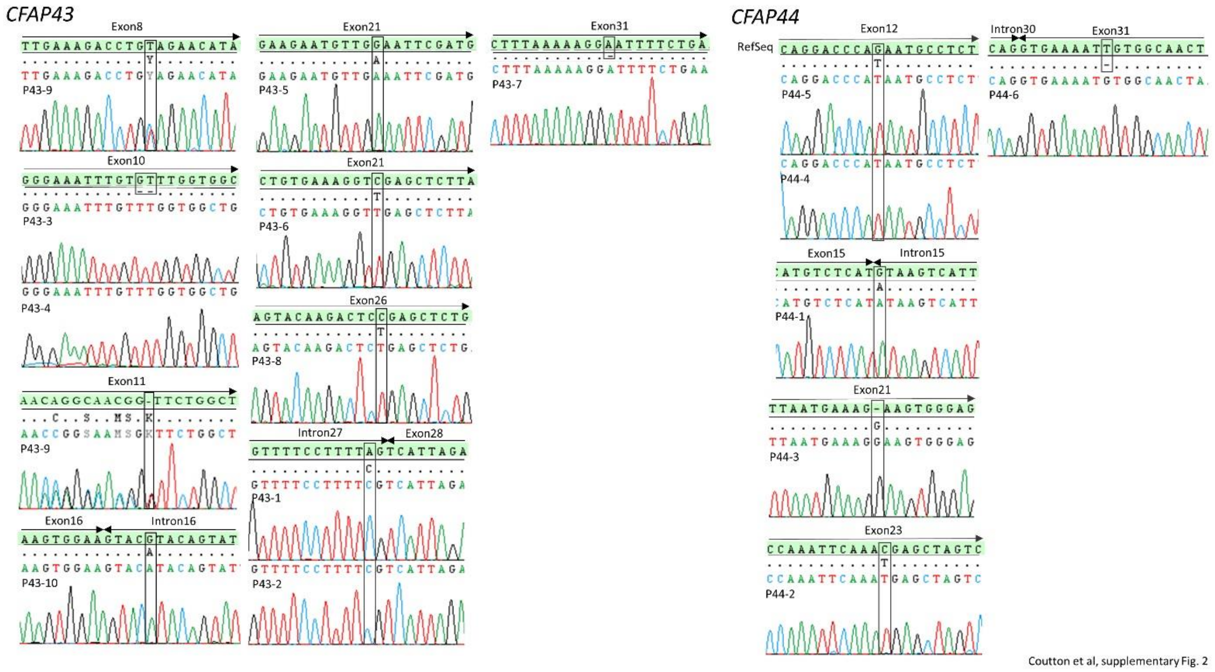

Supplementary Figure 2. Electrophoregrams of Sanger sequencing for all CFAP43 and CFAP44 mutated patients compared to reference sequence.

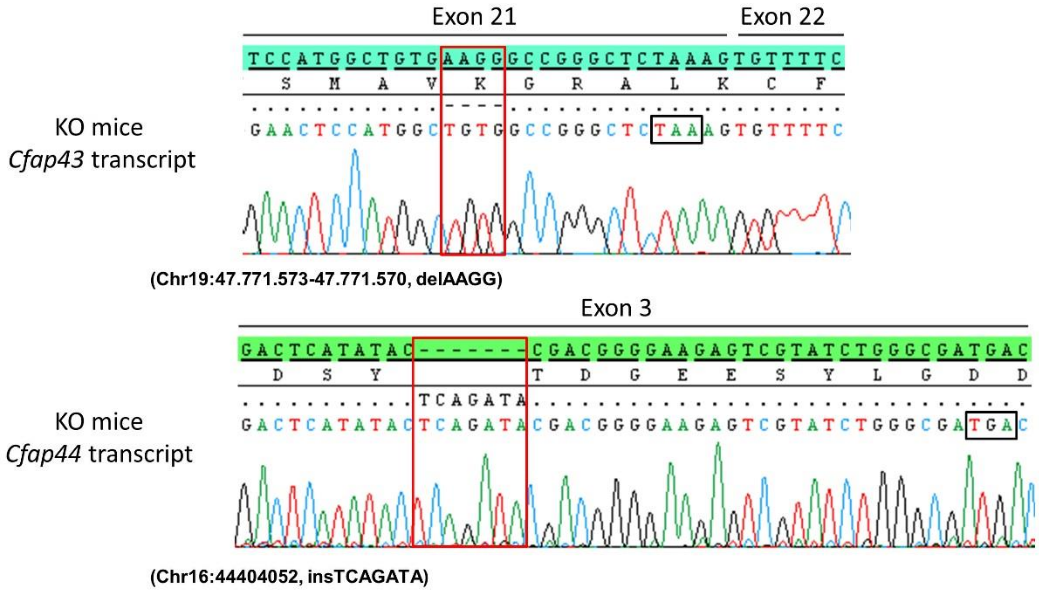

Supplementary Fig. S3

Supplementary Figure 3. Identification of indels in mice generated by CRISPR/Cas9 technology. *Cfap44* and *Cfap43* KO mice were generated by targeting exon 3 and 21, respectively. Sequencing of transcripts obtained from the testis of mutant mice allowed us to identify a frameshift mutation InsTCAGATA in *Cfap44* and delAAGG in *Cfap43* (Red boxes) leading to a premature stop codon (Black box).

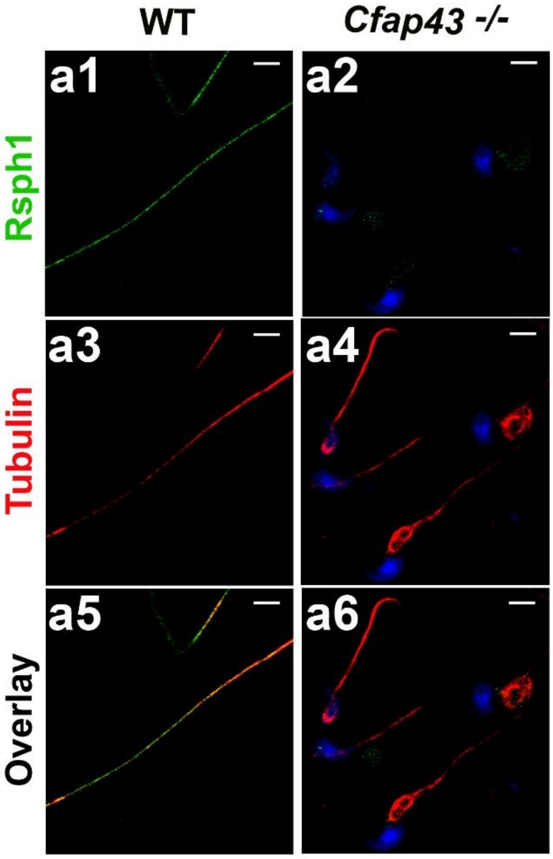

Supplementary Figure 4. Head of radial spoke Rsph1 are absent in sperm from Cfap43$^{-/-}$ males. (a1,a3) Staining of WT sperm with an anti-Rsph1 (**a1**, green) and anti-tubulin (**a3**, red) antibodies. (**a5)** overlay of Rsph1 and tubulin staining. Sperm were counterstained with Hoechst (blue). (**a2, a4, a6)** Similar experiments on sperm from *Cfap43*$^{-/-}$ males. Scale bars = 5 µm.

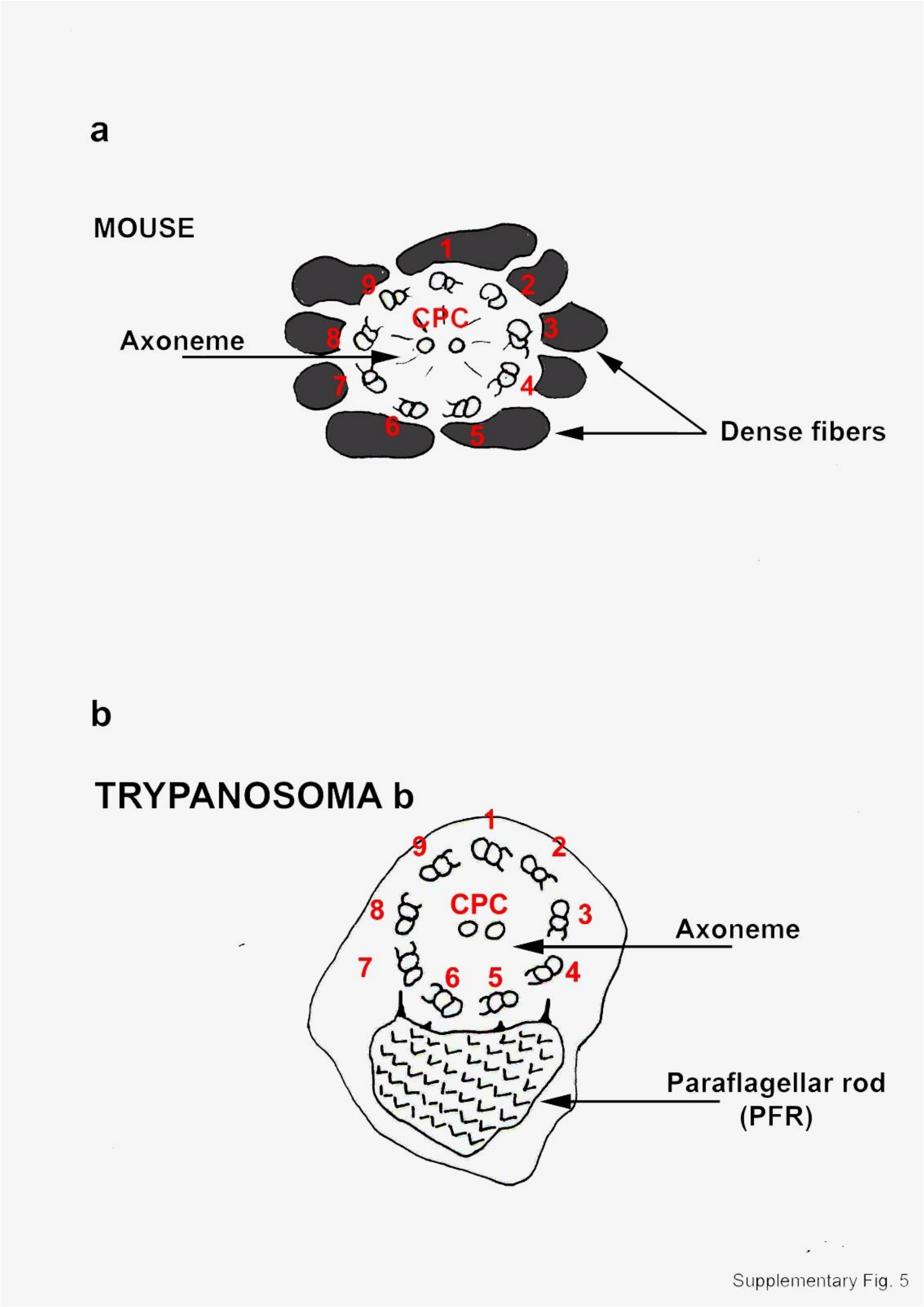

Supplementary Figure 5. Drawing of the ultrastructure of mouse and *Trypanosoma b.* axonemes, showing the annotations of the DMTs.

a. In mouse, drawing representing a section of the midpiece of the flagellum. The mitochondria and the plasma membrane are not represented. The axoneme is surrounded by 9 dense fibers.

b. In *Trypanosoma b.*, drawing representing a section of the flagellum and showing the organization of the axoneme and the PFR.

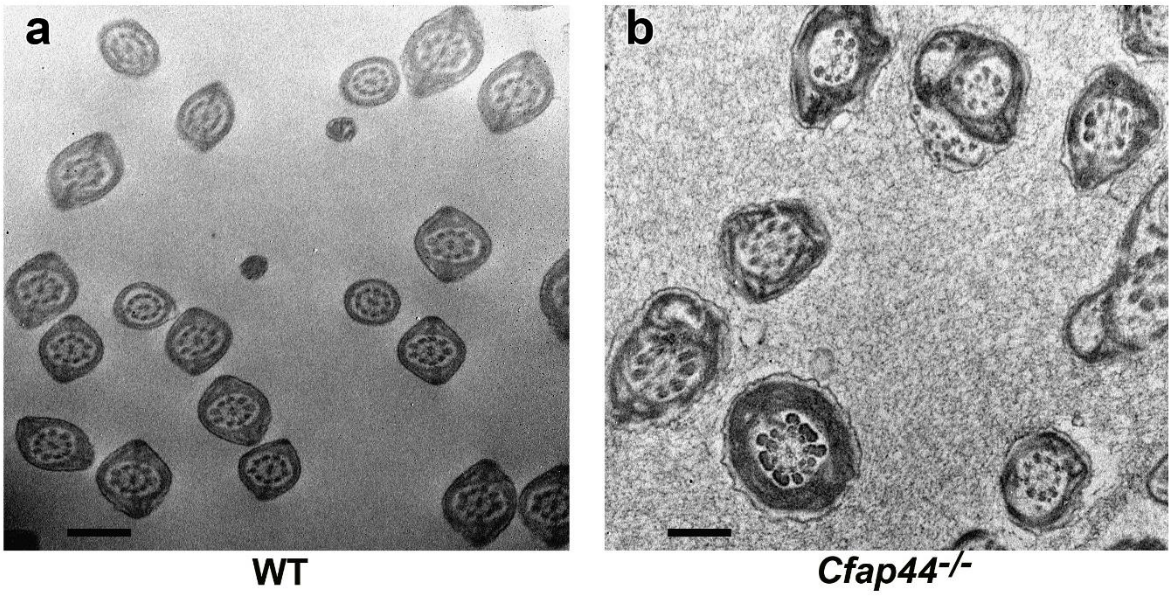

Supplementary Figure 6. Electron microscopy images of the ultrastructure of sperm from WT and Cfap44$^{-/-}$ male at low magnification.

(a) Note the uniform structure of the WT sperm. **(b)** In contrast, in *Cfap44*$^{-/-}$ sperm, the overall shapes of the flagella are heterogeneous. Scale bars = 402 nm.

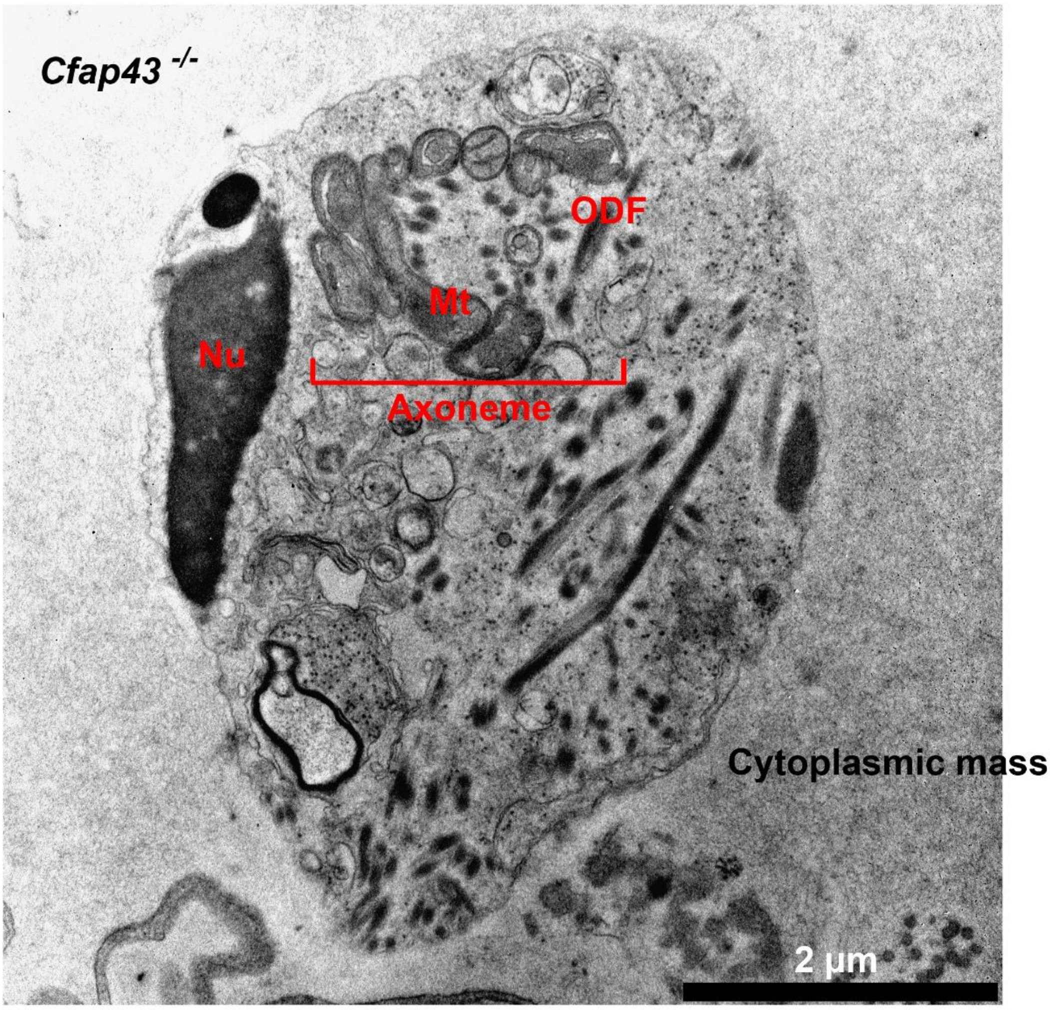

Supplementary Figure 7. TEM of sperm from Cfap43-/- exhibiting a disorganized cytoplasmic mass. Electron microscopy images of sperm from *Cfap43*$^{-/-}$ males show the absence of organized flagellum in short tail sperm. The short tail rather corresponds to an unorganized cytoplasmic mass containing the different components of the flagellum. scale bar = 2 µm.

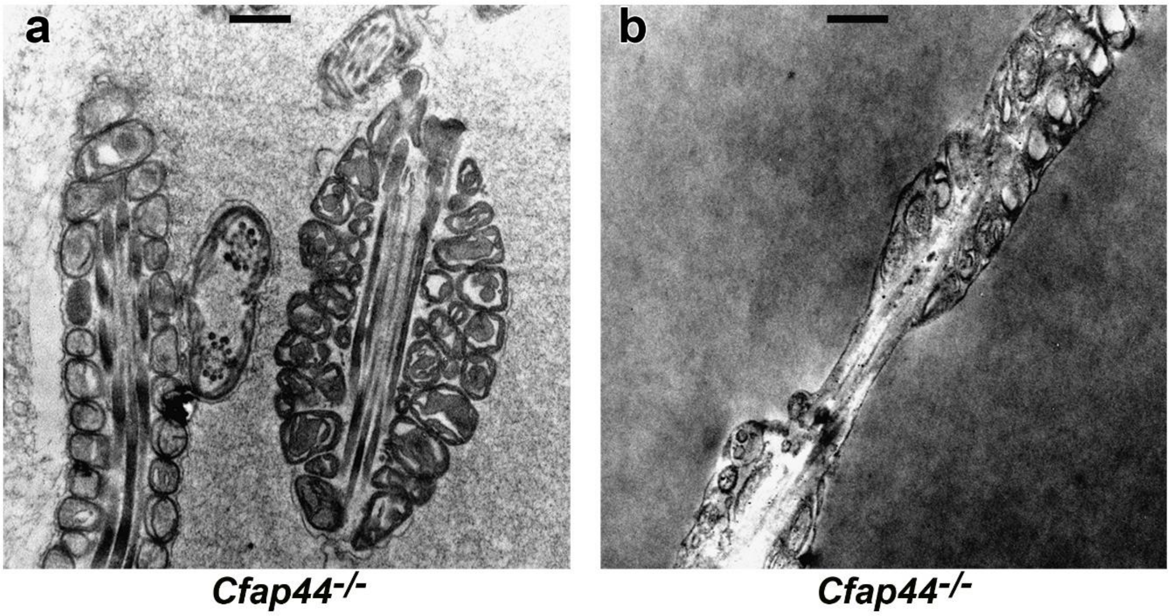

Supplementary Figure 8. Defective mitochondria organization in sperm from Cfap44$^{-/-}$
Longitudinal sections obtained by electron microscopy of sperm from *Cfap44*$^{-/-}$ males showing that the mitochondria are fragmented (**a**) and irregularly layered (**b**). Left image scale bar = 510 nm and right image scale bar = 395 nm.

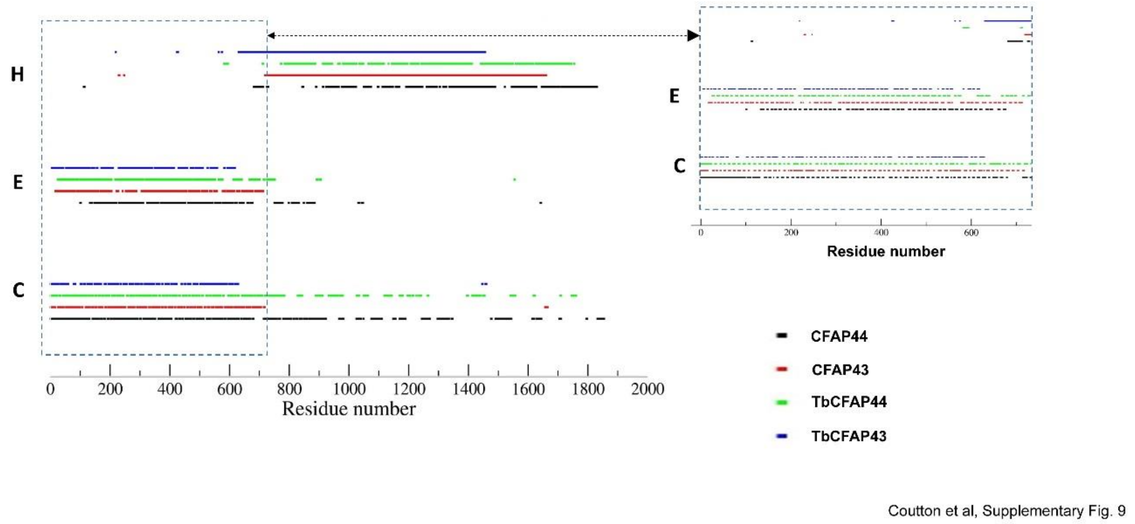

Supplementary Figure 9. Secondary structure prediction. Elements of the secondary structures of the CFAP44, TbCFAP44, CFAP43, TbCFAP43 were predicted by Porter 4.0. C, E and H correspond to coils, extended conformations (β-sheets) and helices (α-helices), respectively. C, E, H are shown for all residues and a detail of the N-ter β-domain is highlighted in the inset.

a

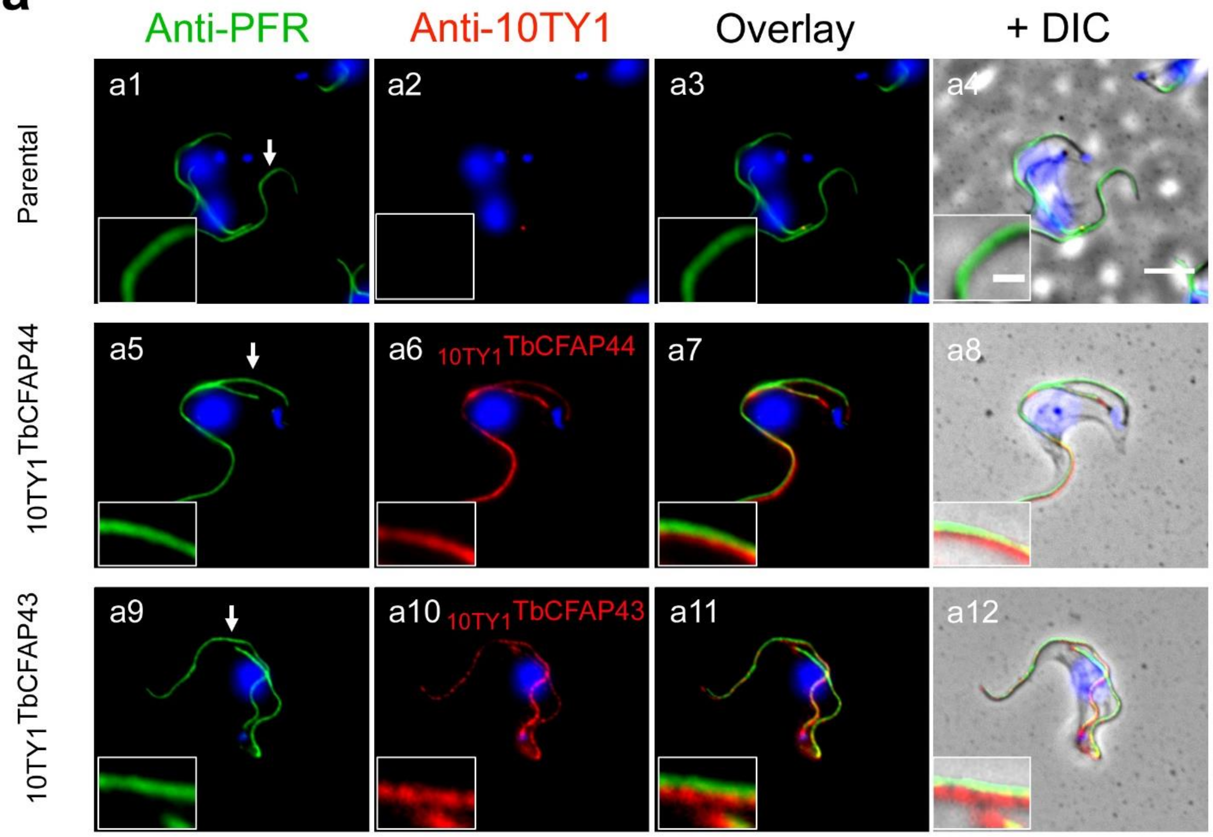
Anti-PFR
Anti-10TY1
Overlay
+ DIC
Parental
10TY1TbCFAP44
10TY1TbCFAP43
a1
a2
a3
a4
a5
a6 10TY1TbCFAP44
a7
a8
a9
a10 10TY1TbCFAP43
a11
a12

b

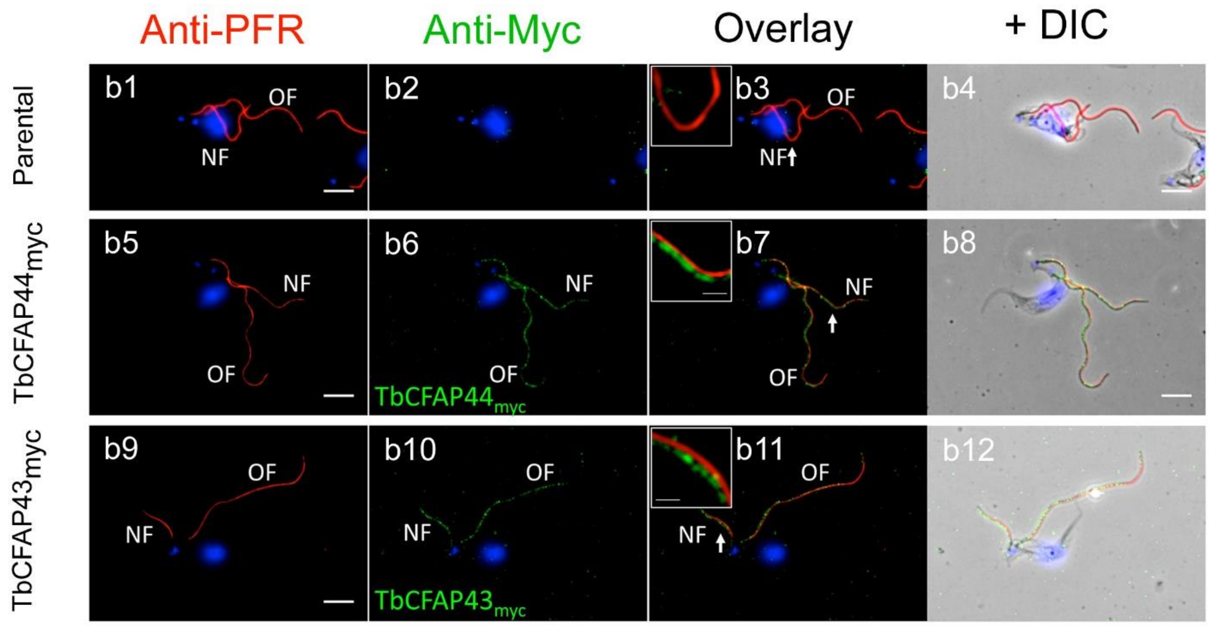
Anti-PFR
Anti-Myc
Overlay
+ DIC
Parental
TbCFAP44myc
TbCFAP43myc
b1
OF
NF
b2
b3
OF
NF
b4
b5
NF
OF
b6
NF
OF
TbCFAP44myc
b7
NF
OF
b8
b9
OF
NF
b10
OF
NF
TbCFAP43myc
b11
OF
NF
b12

Supplementary figure 10. Orthologs of T. brucei TbCFAP44 and TbCFAP43 are flagellar proteins. **(a)** Localization of $_{10TY1}$TbCFAP44 and $_{10TY1}$TbCFAP43 were observed by IF. **(a1-a4)** Immunofluorescence on detergent extracted cells of parental *T. brucei* (non expressing myc-tagged proteins) stained with anti-PFR (green) and anti-TY1 (red) antibodies. No TY1 staining was observed on parental cells, confirming the specificity of the anti-TY1 antibody. **(a5-a8)** Immunofluorescence on detergent extracted cells constitutively expressing 10TY1-tagged TbCFAP44 and non-induced for *TbCFAP43^{RNAi}*. The flagella were labelled with the anti-PFR (green), and 10TY1-tagged protein with anti-TY1 (green). Note the red staining of the entire flagellum showing that TbCFAP44 localisation is restricted to the flagellum. **(a9-a12)** Similar experiments as performed in **a5**-**a8** for TbCFAP43. Insets are enlargement of images of flagella taken from the main panels and display areas indicated by white arrows (scale bar 1 µm). Nuclei and kinetoplasts (mitochondrial genome) are labelled with DAPI (blue). Scale bars represent 5 µm. (b). Similar experiments performed with TbCFAP44$_{myc}$ and TbCFAP43$_{myc}$ proteins and stained with anti-PFR (red) and anti-myc (green) antibodies. An identical localization of both proteins is observed with both tags.

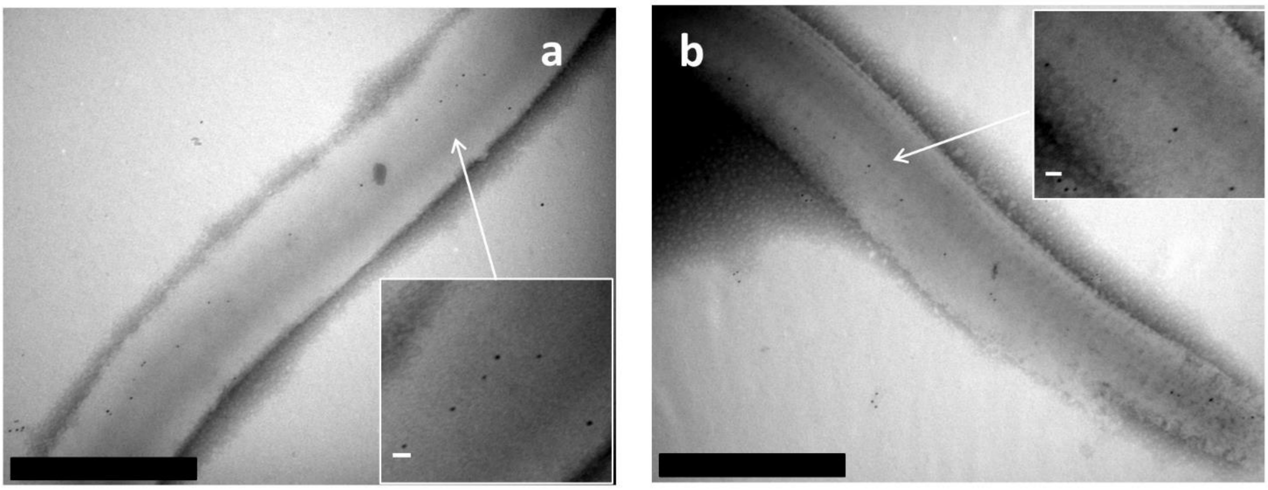

$_{10TY1}$TbCFAP44 $_{10TY1}$TbCFAP43

Supplementary Figure 11. Ultrastructural localization of ^{10TY1}TbCFAP$_{43}$ and ^{10TY1}TbCFAP$_{44}$. Electron immuno-gold labelling of detergent-extracted cytoskeleton cells expressing $_{10TY1}$*TbCFAP44* (a) and of $_{10TY1}$*TbCFAP43* (b). Gold beads size was 6 nm Scale bars represent 500 nm. Insets are enlargement of images of flagella taken from the main panels. Scale bars represent 10 nm.

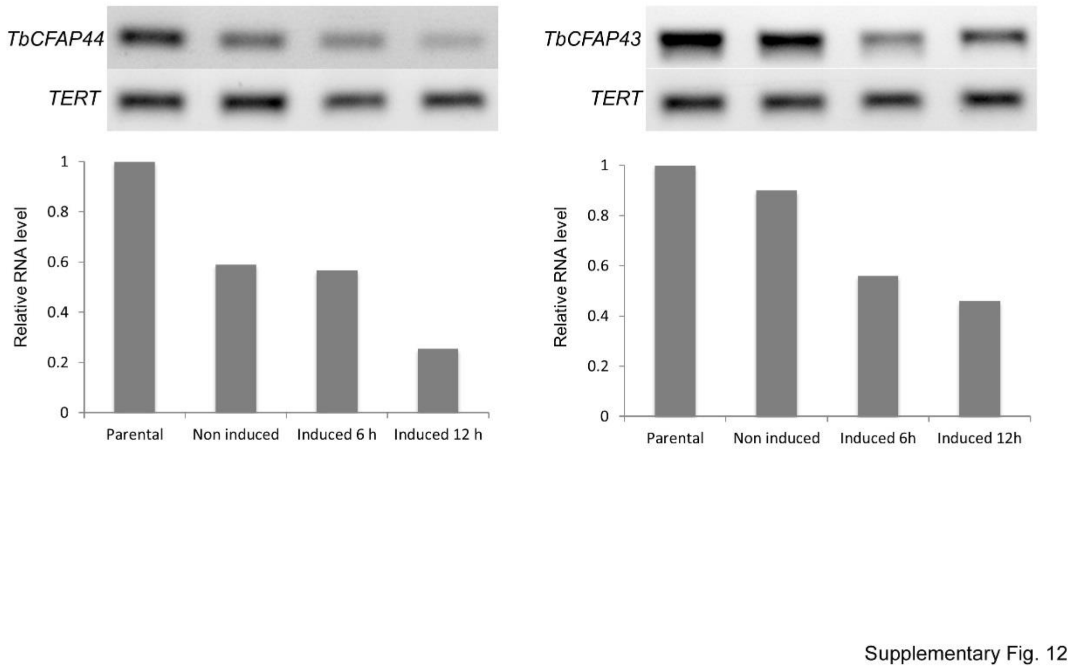

Supplementary Figure 12. Quantification of TbCFAP43 and TbCFAP44 RNAi knockdown. Total RNA was isolated from parental and non-induced cells, and from 6 and 12h RNAi induced cells. mRNA decrease upon RNAi induction was tested by semi-quantitative RT-PCR using primer sets specific to *TbCFAP44* (left panel) or *TbCFAP43* (right panel) and quantified (lower panel) relative to the telomerase reverse transcriptase (TERT) level.

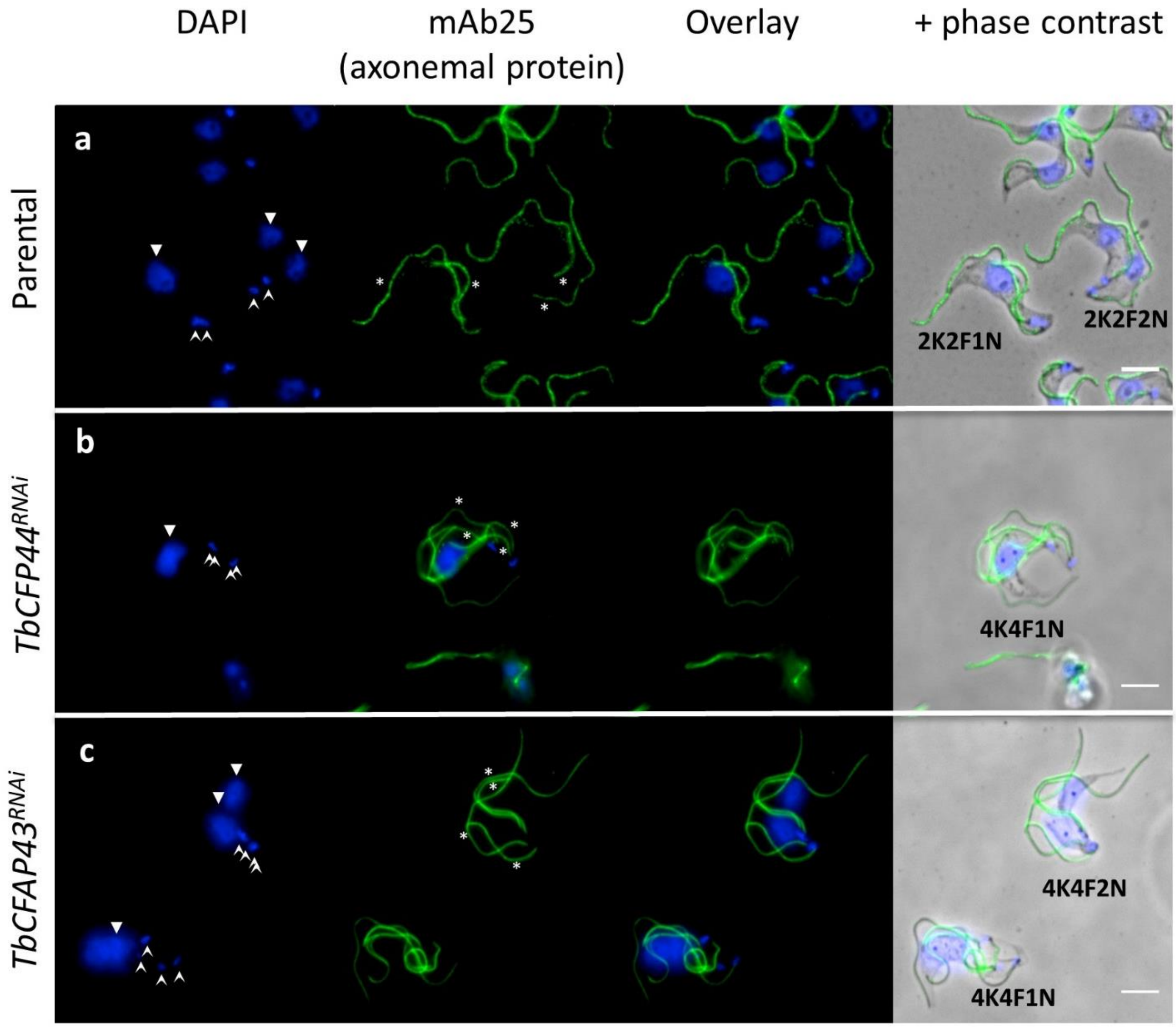

Supplementary figure 13. Knockdown of TbCFAP43 and TbCFAP44 induces cytokinesis defects and produces multiflagellated cells. Parental cells **(a)**, and cells induced 24h for the RNAi of *TbCFPA44* **(b)** and *TbCFAP43* **(c)** were detergent extracted and methanol fixed. The flagella (F, asterisk) were immuno-labelled with mAb25, an anti-axonemal protein (green). Parental cells have 2 flagella whereas mutant cells have 4. The nuclei (N, triangle) and the kinetoplasts (K, arrow head) were labelled with DAPI. Scale bars 5 µm. 4K4F2N means cell with 4 kinetoplasts, 4 flagella and 2 nuclei.

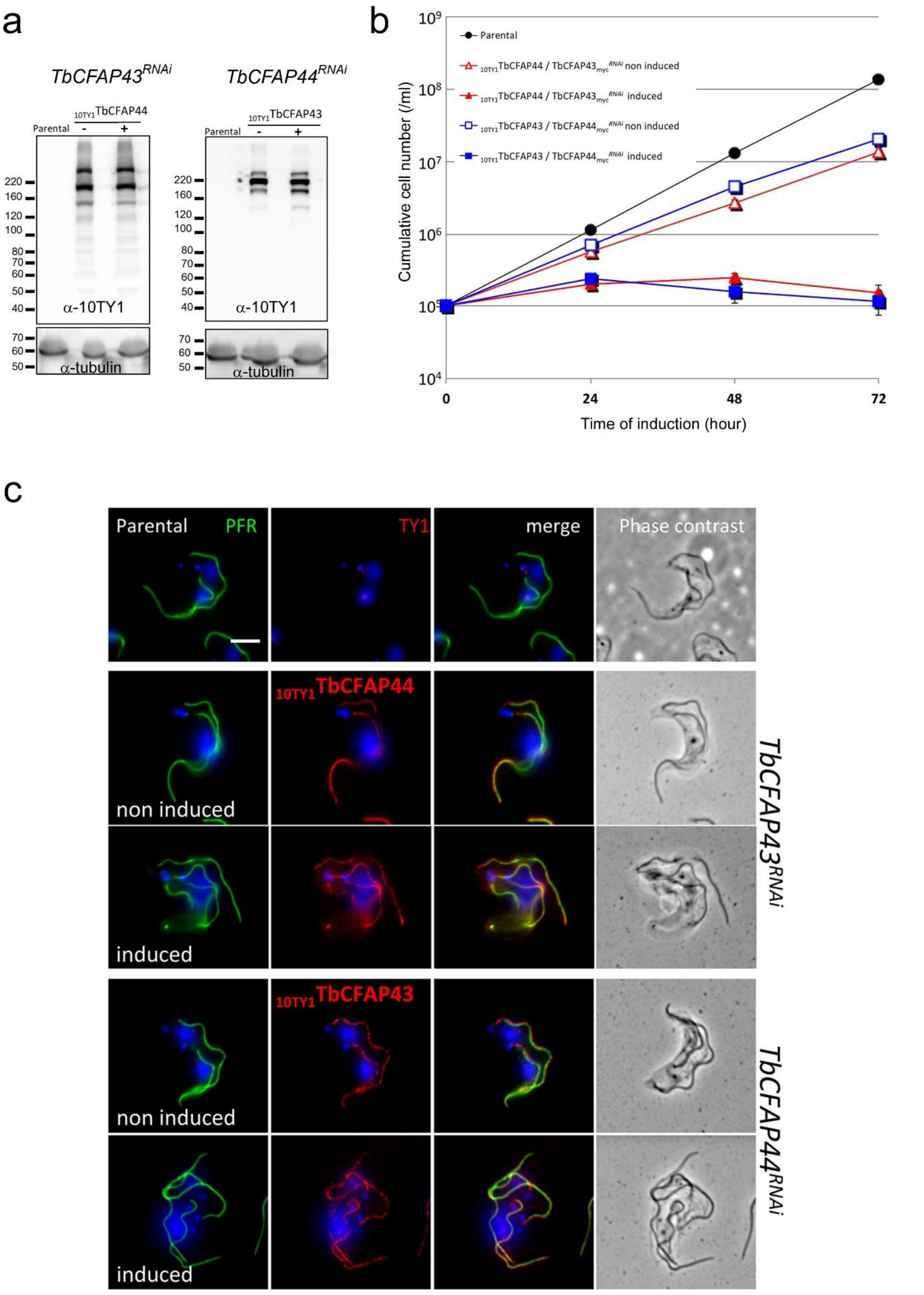

Supplementary Fig. 14

Supplementary Figure 14. Expression of *TbCFAP43* does not rescue cell death induced by knock-down of *TbCFAP44* and vice versa. (a) Western-blot analysis of the expression of endogenously tagged $_{10TY1}$*TbCFAP44* and $_{10TY1}$*TbCFAP43* in the background of the cell line *TbCFAP43*$_{myc}$/*TbCFAP43* RNAi and *TbCFAP44*$_{myc}$/*TbCFAP44* RNAi respectively. RNAi was non induced (-) or induced (+) for 24 h. Expected MM of $_{10TY1}$*TbCFAP44* is 213.7 KDa, and of $_{10TY1}$*TbCFAP43* is 180 KDa. Loading was controled using anti-tubulin. Detergent-extracted cytoskeleton (2.10^7) were loaded on a 6% SDS-PAGE. **(b)** Growth curves for parental cells, and $_{10TY1}$*TbCFAP44*/*TbCFAP43*$_{myc}^{RNAi}$ and $_{10TY1}$*TbCFAP43*/*TbCFAP44*$_{myc}^{RNAi}$ cells, non-induced or induced with tetracycline. Cells were counted every 24 h. The graph represents the cumulative number of cells per ml. Error bars represent the standard error from 3 independent experiments. **(c)** Immunofluorescence on detergent extracted cells of parental *T. brucei* cell, $_{10TY1}$*TbCFAP44*/*TbCFAP43*$_{myc}^{RNAi}$ and $_{10TY1}$*TbCFAP43*/*TbCFAP44*$_{myc}^{RNAi}$ cells (non-induced or induced with tetracycline) and labelled with anti-PFR (green) and anti-TY1 (red) antibodies. Anti-TY1 labelling is still present on all flagella in RNAi induced cells. Nuclei and kinetoplasts (mitochondrial genome) are labelled with DAPI (blue). Scale bars represent 5 µm.

SUPPLEMENTARY TABLES

Supplementary Table 1. Sperm axonemal abnormalities observed by transmission electron microscopy (TEM) in individuals P_{43}-8 carrying mutations in CFAP43 and in individual P_{44}-3 carrying mutations in *CFAP44*.

Individual	Normal (9+2)	Abnormal	CPC defects (9+0)	CPC defects (9+1)	CPC defects (8+1)	CPC and DMTs defects
**CFAP 43 (P_{43}-8)**	4.6*	96.4	81.8	0	0	13.6
**CFAP 44 (P_{44}-3)**	4.8*	95.2	66.7	9.5	0	19
**Control**	100	0	0	0	0	0

Number of section analyzed: 23 sections for fertile control, 21 sections for CFAP44 mutated patient (1 patient) and 22 sections for CFAP43 patient (1 patient).

CPC central pair complexe; DMTs Doublets of microtubules

* Peri-axonemal structures (i.e. longitudinal columns) were mislocalized in all cases; a defect also observed in 4.3% of control sections.

Supplementary Table 2. Absence of CFAP43 or CFAP44 modulate intensities of staining of different axonemal substructures in mouse and human flagella.

The following antibodies were used to identify sub-components of the axoneme: α-RSPH1 and α-RSPH4A for radial spokes, α-GAS8 for N-DRC, α-DNALI1 for IDA, α-DNAI2 and α-DNAH5 for ODA, α-SPAG6 and α-SPEF2 for CP and α-AKAP4 for fibrous sheath. ++ intense staining, + intermediate staining, +/- weak

**HUMAN**	**Antibody**	**Fertile control**	***CFAP43 mutated* patients**	***CFAP44 mutated* patients**
**Radial spoke**	α-RSPH1	++	atypical	+/--
	α-RSPH4A	+	atypical	+/--
**Central pair complex**	α-SPAG6	++	-	Atypical weak
**N-DRC**	α-GAS8	+	+	+
**IDA**	α-DNALI1	++	++	++
**ODA**	α-DNAI2	++	++	++
**Fibrous sheath**	α-AKAP4	++	++	++

Supplementary Table 2 (continued)

MOUSE	Antibody	WT	*Cfap43*$^{-/-}$	*Cfap44*$^{-/-}$
**Radial spoke**	α-RSPH1	++	-	++
	α-RSPH4A	++	-	++
**Central pair complex**	α-SPEF2	+	-	+
**N-DRC**	α-GAS8	+	+	+
**IDA**	α-DNALI1	++	++	++
**ODA**	α-DNAH5	++	++	++

Supplementary Table 3. Protein folds found in the 4 target sequences.
WD40 folds are found for all N-ter domains of the proteins using the Superfamily program with very good confidence (e-value corresponds to the probability of finding such folds by chance in the sequence).

Protein	Family	Domains (aa number)	E-value
CFAP44	WD40 repeat-like<br>WD40 repeat-like<br>WD40 repeat-like	234-285,379-398,460-632<br>565-661,740-890<br>146-396	2.93e-40<br>3.71e-21<br>2.23e-20
CFAP43	WD40 repeat-like	110-216,347-503,626-713	3.4e-30
Prot-TbCFAP44	WD40 repeat-like<br>WD40 repeat-like	75-249,276-338,392-462<br>389-567,672-748	1.46e-36<br>1.47e-15
Prot-TbCFAP43	WD40 repeat-like<br>Tricorn protease domain 2	228-279,319-466,528-616<br>34-281	2.0e-12<br>1.8e-08

Supplementary Table 4. Primers used for *CFAP43* and *CFAP44*'s patients Sanger sequencing verification.

Primer name	Primer sequence (5'-3')	Amplicon Size (bp)
CFAP43-Ex8-F	AGGACAGTGCATCTATGCAAAC	516 bp
CFAP43-Ex8-F	TGCAGCATACTCTGCTCCTC	
CFAP43-Ex10-F	TTCAAAAACAACTGCCACCA	409 bp
CFAP43-Ex10-R	GCACACTGGAAAGGGAGGTA	
CFAP43-Ex11-F	TCCTGTGCCATCTTATGCAG	533 bp
CFAP43-Ex11-R	TGCAAGGACAGACCTGGGTA	
CFAP43-Ex16-F	AAGCCGTGTGGTAGAATTGC	464 bp
CFAP43-Ex16-R	ATCCTAAACCTGCCCAATCC	
CFAP43-Ex21-F	AGTGCAAGGTGGCTCAAAGT	439 bp
CFAP43-Ex21-R	GACTGAGCACAGAGGGAAGG	
CFAP43-Ex26-F	TCTAATGTTGGGTGGGAAGC	386 bp
CFAP43-Ex26-R	GCCTCCTCACTTAGCCTCCT	
CFAP43- Ex28-F	CAAGTGGGAGCATCTCTGGT	434 bp
CFAP43- Ex28-R	GTAAAGGGGAAGGTGCACAA	
CFAP43-Ex31-F	AGGTTTCCAAACCAAGTTGC	382 bp
CFAP43-Ex31-R	ACCGTGCTTACACAGATCCTT	
CFAP44-Ex12-F	ACTTGCGCAAAATCACACAG	421 bp
CFAP44-Ex12-R	GGAAAATCCCCAAATGCTCT	
CFAP44-Ex15-F	AATGGGGAAAATTGAGCAAG	401 bp
CFAP44-Ex15-R	CTGACCCTCACTGCAGGAAC	
CFAP44-Ex21-F	TTGAAACAGAGCCAATTCCA	530 bp
CFAP44-Ex21-R	GATGCCCAAAATAAATGGCTA	
CFAP44-Ex23-F	CAATCCAACTTTCATGACATCC	484 bp
CFAP44-Ex23-R	AGAGGGGCAAGAGATTGAGC	
CFAP44-Ex31-F	GGACATGTCTTCTGGGCTCT	443 bp
CFAP44-Ex31-R	TGAAAAGCTTGGGTGTGATG	

Supplementary Table 5. Primers used for RT-qPCR of *CFAP43 and CFAP44* in human and mouse.

Primer names	Primer Sequence (5'-3')	Tm (C°)
CFAP43_5F<br>*CFAP43_6R*	CTGGTAGGCAAAGAGGCAGA<br>TCACAGCCAATGTACAAGTC	60
Cfap43_2F<br>*Cfap43_3R*	ATGGCAACTAATGTGCCCAGT<br>GCAAAGTAGAGTGTAGTCCAGGA	60
CFAP44_5F<br>*CFAP44_6R*	CTACCTGCGAAGTAGCAGTG<br>CGAAGGACTCTGTATGGTCT	60
Cfap44_15F<br>*Cfap44_16R*	TTGCCAGTTGATGTGGTCTC<br>ACACGTTCGGGATTGCAAAC	60
ACTB_F<br>*ACTB_R*	CCAACCGCGAGAAGATGA<br>CCAGAGGCGTACAGGGATAG	60
Actb_F<br>*Actb_R*	ACCAGAGGCATACAGGGACA<br>CTAAGGCCAACCGTGAAAAG	60

Supplementary Table 6. List of primary antibodies used in immunofluorescence experiments in human, mouse and *Trypanosoma*

Primary antibodies	Reference	Species	Protein localization	Dilution
**Human experiments**				
DNAI2	Abnova H00064446-M01	mouse	ODA	1/400
DNALI1	Sigma-Aldrich® HPA028305	rabbit	IDA	1/100
RSPH1	Sigma-Aldrich® HPA017382	rabbit	RS, head	1/100
RSPH4A	Sigma-Aldrich® HPA031196	rabbit	RS, head	1/100
GAS8	Sigma-Aldrich® HPA041311	rabbit	N-DRC	1/100
SPAG6	Sigma-Aldrich® HPA038440	rabbit	CPC	1/500
AKAP4	Sigma-Aldrich® HPA020046	rabbit	FS	1/100
Acetylated tubulin	Sigma-Aldrich® T7451	mouse	Microtubules	1/2000
**Mouse experiments**				
Rsph1	Sigma-Aldrich® HPA017382	rabbit	RS, head	1/100
Rsph4a	Sigma-Aldrich® HPA031196	rabbit	RS, head	1/50
Gas8	Sigma-Aldrich® HPA041311	rabbit	N-DRC	1/100
Spef2	Sigma-Aldrich® HPA040343	rabbit	CPC	1/1000
Dnali1	Sigma-Aldrich® HPA028305	rabbit	IDA	1/100
Dnah5	Sigma-Aldrich® HPA037470	rabbit	ODA	1/100
Mpc1l	Described in (1)	rabbit	Mitochondria	1/500
Acetylated tubulin	Sigma-Aldrich®® T7451	mouse	Microtubules	1/500
***T brucei* experiments**				
myc	Santa Cruz monoclonal 9E10, sc-40	mouse	Myc-tagged proteins	1/10
PFR2	Gift of Dr N. Biteau (2)	rabbit	PFR / flagellum	1/2000; 1/400 (STED)
mAb25 (TbSAXO)	Described in (3)	mouse	Microtubules/axoneme	1/5
BB2 (TY1)	Described in (4)	mouse	TY1-tagged proteins	1/500 (IF); 1/10 (iEM)
TAT1 (tubulin)	Described in (4)	mouse	Cytoskeleton / flagellum	1/50 (IF); 1/10 (iEM)
L8C4 (PFR2)	Described in	mouse	PFR / flagellum	neat

Abbreviations are as follows : ODA, Outer Dynein Arms ; IDA, Inner Dynein Arms ; RS, Radial Spokes ; FS, Fibrous sheath ; CPC, Central Pair Complex;

(1) Vanderperre et al, (2016) MPC1-like Is a Placental Mammal-specific Mitochondrial Pyruvate Carrier Subunit Expressed in Postmeiotic Male Germ Cells.J Biol Chem. 2016 Aug 5;291(32):16448-61. doi: 10.1074/jbc.M116.733840.

(2) Laboratoire de Microbiologie Fondamentale et Pathogénicité UMR5234-CNRS F-33076 BORDEAUX

(3) D. Dacheux et al., (2012) A MAP6-Related Protein Is Present in Protozoa and Is Involved in Flagellum Motility," PLoS One 7 (2): e31344, doi:10.1371/journal.pone.0031344.

(4) Bastin et al., "A Novel Epitope Tag System to Study Protein Targeting and Organelle Biogenesis in Trypanosoma Brucei," *Mol Biochem Parasitol* 77, no. 2 (1996): 235–39.

(5) Linda Kohl, Trevor Sherwin, and Keith Gull, "Assembly of the Paraflagellar Rod and the Flagellum Attachment Zone Complex During the Trypanosoma Brucei Cell Cycle," *Journal of Eukaryotic Microbiology* 46, no. 2 (March 1, 1999): 105–9, doi:10.1111/j.1550-7408.1999.tb04592.x.

Supplementary Table 7. Endogenous tagging and RNAi primer sequences for *T. brucei* experiments

3xmyc C-terminal endogenous tagging primers
TbCFAP44
5'-
GGAAATTGATGCACTGAGAACAGAAGTTGCACTCCTACGTACGAAGGGTGGACACGTGTATGCCGCTGCTAT
GGCAGCCGGCAGGggtaccgggccccccctcgag-3'

5'-
CACTCCTTCCCAAATACATACTGGTATTGAAGCGCTTCGCTGTTCGTGATGGTGCCAAGGCACAACATAGAGAG
AAACTAGCAGCtggcggccgctctagaactagtggat-3'

TbCFAP43
5'-
ACTTGTTCGCCTTAAAAACGAGGTAGATCGCTTGCGTGAGGCCACATTTCCCTCATTTGCTGTTGTTACGAGGA
GGACCGCCAGAggtaccgggccccccctcgag-3'

5'-
CTTCCTTCTGCCACTTGTCCGAATAACGCCGACTTCTGCCTCACTTGCCCCCCCTCGCCAGTGTCAGCAGGGCAC
CGCCAtggcggccgctctagaactagtggat-3'

RNAi primers:
TbCFAP44 (target bp 1880-2309 within the ORF)
5'-GCCGGCCGCTCTAGAgggccggtggagtagagct-3'
5'-TAAGCTTGCTCTAGAggttggagttgggcaacagg-3'

TbCFAP43 (target bp 131-596 within ORF)
5'-GCCGGCCGCTCTAGActtgtgaaggccgc-3'
5'-TAAGCTTGCTCTAGAgaccctcagtgccccatacg-3'

RT-PCR primers
TbCFAP44
5'-GGCTATTTTCGCTTCTGGCG -3'
5'-GCTTCGTCACAGCGTTGTTT -3'

TbCFAP43
5'-AGAGACTGAACAGGCGCTTC -3'
5'-TCGCTCACCATAGCGTTCTC -3'
TbTERT
5'-GAGCGTGTGACTTCCGAAGG-3'
5'-AGGAACTGTCACGGAGTTTGC -5'

Supplementary Table 8. Guide RNA sequences for CRISPR/Cas9 mice generation.

Gene	Exon targeted	Guide RNA sequence (5’-3’)
Cfap43	exon 2	AATGTGCCCAGTGAAGTCG
	exon 21	CTCCATGGCTGTGAAGGGC
Cfap44	exon 3	CAGCTGACTCATATACCGA
	exon 15	AGAAAGAATACAAGCCGA

Supplementary Table 9. Primers for CRISPR/Cas9 mice genotyping.

Gene	Method	PCR Primer Name	PCR Primer sequence	Tm (°C)
Cfap43	Sanger sequencing	Wdr96m_2F	CAAAGATGGGTGCAAGGATT	56
		Wdr96m_2R	AGCAGGGGGAAGAATGAACT	56
		Wdr96m_21F	GTGCAGGGGAATCCTGTAAA	58
		Wdr96m_21R	AGCAGGGGGAAGAATGAACT	59
	HRM	96HRM_21F	TGTGGAGATGCACAACTTGG	58
		96HRM_21R	GTCGGAGGCTGTGTGTCTCT	61
Cfap44	Sanger sequencing	Wdr52mT1_3F	TCTATACACGAAGCTTCACAAGACA	60
		Wdr52mT1_3R	AGAAACAGAATGTAGAACCCCTGA	59
	HRM	52HRM_3F	CGAAGCTTCACAAGACACCA	58
		52RHRM_3R	GCAGGAGCCTGAACGTACTC	60

Supplementary Table 10. List of primers used for RT-PCR experiments for *Cfap43 and Cfap44.*

Primer name	Exons	Primer sequence (5'-3')	Tm
Cfap43_E20-21F	20-21	GGAGGAAGTAGCGAAGATAA	60
Cfap43_E22R	22	CAACTCCTGTAGCTCCTCTT	60
Cfap44_E2F	2	ATATGAAGGAGCCAGATGAC	60
Cfap44_E3R	3	CGTACTCTTGAGATGAATGC	60
