# Supplementary material for: Mutations in CFAP43 and CFAP44 cause male infertility and flagellum defects in Trypanosoma and human
Source: Nat Commun. 2018 Feb 15;9:686. doi: 10.1038/s41467-017-02792-7 (PMC5814398; doi:10.1038/s41467-017-02792-7)
Supplement: Supplementary file 3 — Description of Additional Supplementary Files [file 41467_2017_2792_MOESM3_ESM.pdf]

File Name: Supplementary Movie 1
Description: Video showing the defective flagellum beat of sperm from *Cfap44* $^{-/-}$ males.

File Name: Supplementary Movie 2
Description: Video showing normal flagellum beat of sperm from *Cfap44*$^{+/-}$ heterozygotes males.

File Name: Supplementary Movie 3
Description: Video showing normal flagellum beat of parental (WT) BSF *T. brucei.*

File Name: Supplementary Movie 4
Description: Video showing flagellum beat of CFAP43-RNAi induced *T. brucei (BSF).*

File Name: Supplementary Movie 5
Description: Video showing flagellum beat of CFAP44-RNAi induced *T. brucei (BSF).*
